# Application of Stem Cells in the Oral and Maxillofacial Region

**DOI:** 10.1155/2020/2421453

**Published:** 2020-04-13

**Authors:** Toru Ogasawara, Edward Chengchuan Ko, Jiashing Yu

**Affiliations:** ^1^The University of Tokyo, Tokyo, Japan; ^2^Kaohsiung Medical University, Kaohsiung, Taiwan; ^3^National Taiwan University, Taiwan

## 1. Introduction

Conditions such as trauma-induced bone or cartilage defects and tumor or congenital defects are common in the oral and maxillofacial region. To repair irreversible skeletal damage or defects, bone grafts are the current gold standard. However, problems such as a shortage of bone graft material and donor-site morbidity affect the availability of bone grafts. It is also still difficult to restore salivary glands that have been severely damaged by radiation therapy and to counteract the neurodegeneration induced by trauma or surgery. In addition, due to the limited self-healing ability of the teeth, dental caries is treated by fillings (e.g., composite resin) or crowns, and missing teeth are replaced through dental bridges, removable dentures, or dental implants.

It is thus necessary to establish new treatment strategies for these conditions. One of the most effective strategies is to introduce stem cell-based tissue engineering technology in the oral and maxillofacial region. Accordingly, numerous studies have been conducted and many promising results have been reported. Stem cell-based tissue engineering therapy in this region of the body remains challenging to perfect, however.

The cutting-edge review by J.-Q. Liu et al. emphasized four bone sites (growth plate, perivascular areas, periosteum, and cranial suture) as possible sources of skeletal stem cells (SSCs) and evaluated these cells from a SSC perspective. To make the best use of SSCs, they considered it necessary to clarify the mechanism underlying their fate commitment.

X. Xu et al. evaluated the effect of local application of Semaphorin 3A (Sema3A) combined with adipose-derived stem cell sheets and anorganic bovine bone granules in type 2 diabetes mellitus (T2DM) rats. Their results suggested that this combination can be useful to improve bone healing for T2DM patients.

T. Zhou et al. comprehensively reviewed roles of dental follicle cells (DFCs) in tooth development as well as such characteristics of DFCs as their multilineage differentiation, immunosuppression capability, excellent amplification ability, and tissue engineering potential. They concluded that DFCs can act as groups of excellent cells in future cell-based treatment for tissue repair and regeneration.

C. C. G. Pinheiro et al. studied the osteogenic potential of three types of stem cells (umbilical cord, orbicularis oris muscle, and deciduous dental pulp), aiming at alveolar cleft bone tissue engineering. Their results suggested that dental pulp and orbicularis oris muscles are the best sources of mesenchymal stem cells (MSCs) for bone tissue engineering for cleft lip and palate (CLP) patients. The review by V. Wu et al. presented and discussed the advancement of stem cell application, vascularization, and bone regeneration in the oral and maxillofacial region, with an emphasis on the human jaw. In addition, they proposed new strategies to improve the current techniques, which may lead to feasible clinical applications.

A biomaterial-based approach is one of the technical advances shown to improve both cell engraftment and survival after transplantation. In their original research article, S. Wu et al. evaluated a chitosan/*β*-glycerophosphate (CS/*β*-GP) hydrogel as a vascular endothelial growth factor- (VEGF-) sustained release system and explored its effects on dental pulp stem cells (DPSCs). They hypothesized that thermosensitive chitosan hydrogel could effectively deliver VEGF protein in a sustained release pattern to stimulate differentiation and mineralization of DPSCs.

The original research article by A. Wofford et al. demonstrated that xenogeneic human adipose tissue-derived MSCs, which were delivered to and contained at the bone injury site via a bioinert scaffold, promoted enhanced regeneration of maxillary alveolar tooth defects in rats.

Muscle regeneration is also one of the important topics in the oral and maxillofacial region. With the goal of providing a therapeutic basis for the repair of obstructive sleep apnea (OSA) upper airway injury, L.-Y. Zhu et al. studied the function of genioglossus (GG) muscles and muscle stem cells (MuSCs). Their results highlighted the important role of p53/p21 on the GG muscle during the aging process.

In summary, this special issue encompasses both comprehensive reviews and original research articles highlighting advances in stem cell and biomaterial research relative to the regeneration of the oral and maxillofacial region. We sincerely hope that the articles published in this special issue can help researchers to better understand the recent trends in the application of stem cells in the oral and maxillofacial region.

